# Health and life insurance-related problems in very long-term cancer survivors in Germany: a population-based study

**DOI:** 10.1007/s00432-021-03825-x

**Published:** 2021-10-13

**Authors:** Melissa S. Y. Thong, Daniela Doege, Linda Weißer, Lena Koch-Gallenkamp, Heike Bertram, Andrea Eberle, Bernd Holleczek, Alice Nennecke, Annika Waldmann, Sylke Ruth Zeissig, Ron Pritzkuleit, Michael Schlander, Hermann Brenner, Volker Arndt

**Affiliations:** 1 Unit of Cancer Survivorship, Division of Clinical Epidemiology and Aging Research, German Cancer Research Center (DKFZ), PO Box 101949, 69009 Heidelberg, Germany; 2 Division of Clinical Epidemiology and Aging Research, DKFZ, Heidelberg, Germany; 3 Cancer Registry of North Rhine-Westphalia, Bochum, Germany; 4 Bremen Cancer Registry, Leibniz Institute for Prevention Research and Epidemiology - BIPS, Bremen, Germany; 5 Saarland Cancer Registry, Saarbrücken, Germany; 6 Hamburg Cancer Registry, Hamburg, Germany; 7 Institute for Social Medicine and Epidemiology, University of Lübeck, Lübeck, Germany; 8 Cancer Registry of Rhineland-Palatinate, Mainz, Germany; 9 Cancer Registry of Schleswig-Holstein, Lübeck, Germany; 10 Division of Health Economics, German Cancer Research Center (DKFZ), Heidelberg, Germany; 11 Division of Preventive Oncology, DKFZ and National Center for Tumor Diseases (NCT), Heidelberg, Germany; 12 German Cancer Consortium (DKTK), DKFZ, Heidelberg, Germany

**Keywords:** Financial toxicity, Insurance problems, Cancer survivors, Population-based

## Abstract

**Purpose:**

Limited research suggests that cancer survivors have problems with insurance. Our study aimed to gain insight into the proportion of very long-term (14–24 years post-diagnosis) survivors of breast, colorectal, and prostate cancers who had problems with health (HI) and life (LI) insurance.

**Methods:**

We used data from CAESAR (CAncEr Survivorship—A multi-Regional population-based study). Participants completed questions on change in insurance providers since cancer diagnosis, problems with requesting (additional) HI or LI, and how potential problems were resolved. We conducted logistic regression to determine factors associated with change in statutory HI.

**Results:**

Of the 2714 respondents, 174 (6%) reported having changed HI providers. Most switched between different statutory HI providers (86%), 9% from statutory to private, and 5% from private to statutory. Respondents who changed statutory HI providers were more likely to be prostate cancer survivors (OR 2.79, 95% CI 1.01–7.68) while being ≥ 65 years at time of diagnosis (OR 0.58, 95% CI 0.35–0.96) and having ≥ 2 comorbid conditions (OR 0.61, 95% CI 0.40–0.92) were associated with reduced odds for change. Problems in changing HI were minimal and were resolved with additional contribution. Of the 310 respondents who tried to get LI, 25 respondents reported having difficulties, of whom the majority had their request rejected.

**Conclusion:**

Most cancer survivors did not change their HI nor tried to buy LI after cancer diagnosis. Problems with changing statutory HI were generally resolved with additional contribution while the main problem encountered when buying LI was rejection of request.

**Supplementary Information:**

The online version contains supplementary material available at 10.1007/s00432-021-03825-x.

## Introduction

The financial implications of cancer can extend beyond treatment costs to potential loss of income due to work changes or out-of-pocket costs. Research suggests that cancer survivors, even those living in countries with mainly publicly funded healthcare systems such as Germany, can face financial challenges from cancer (Buttner et al. 2019; Longo et al. 2020). Cancer survivors also have to contend with problems with insurance due to history of cancer (Stewart et al. 2001), an issue of particular importance for younger adult cancer survivors (Kuhlthau et al. 2016; Naughton et al. 2020; Tangka et al. 2020). Among older Dutch and US long-term (> 5 years post-diagnosis) cancer survivors, the prevalence of having problems with insurance ranged from 1 to 6% for health insurance, and 2 to 62% for life insurance (Hastert et al. 2018; Mols et al. 2012; Tamminga et al. 2016). Younger age at diagnosis, shorter time since diagnosis, and lower household income were associated with being denied insurance in a cohort of older (50–79 years at diagnosis) female long-term cancer survivors (Hastert et al. 2018).

Health insurance coverage could be associated with health-related quality of life (HRQOL), although research is limited and results are inconsistent. A US study reported that prostate cancer survivors with Medicare insurance or no health insurance reported significantly poorer HRQOL at 2 year follow-up when compared with survivors with health maintenance organizations insurance (Penson et al. 2001), and age could be associated with these differences (Sadetsky et al. 2008). In contrast, studies of on-treatment cancer survivors from Hong Kong or Southeast Asia found no association between insurance status and HRQOL (Chiu and Yang 2019; Kimman et al. 2015). However, these studies reported on healthcare systems that are vastly different to that of Germany.

Germany’s healthcare system is considered the least restrictive and most consumer-oriented in Europe (Health Consumer Powerhouse 2019). Health insurance coverage is compulsory and the majority of the population (~ 88% as of 2020) is covered by statutory health insurance (sHI) (Busse and Blümel 2014; Federal Ministry of Health 2020). Individuals whose gross annual income exceeds the limit set for sHI can choose for private health insurance (pHI). Traditionally, there was little competition between sHI providers as the choices were limited to either local/regional providers or those specific to a profession/professional group. As of 1996, the sHI market was liberalized and members could have free choice in selecting a provider (Busse et al. 2017). However, the proportion of cancer survivors who changed health insurance providers and the problems they may thereby encounter is understudied. In contrast to health insurance, life insurance coverage in Germany is voluntary. The German insurance market is a robust 6th largest in the world, accounting for over 88 million life insurance contracts in 2017 (Gesamtverband der Deutschen Versicherungswirtschafte e.V. 2018). However, the actual number of persons with life insurance is unknown. Research into the scope of problems either with health or life insurance in long-term survivors of cancer is limited, and especially so within the German context.

With almost 500,000 annual new cases of cancer and nearly 4.5 million cancer survivors (including 3 million long-term cancer survivors) (Robert Koch Institute 2019), having a better picture of potential financial problems faced by cancer survivors could be important for health policy makers. Therefore, the aims of the current study were to gain insight into: (1) change in health insurance provider, (2) proportion of problems encountered in changing health insurance or getting life insurance, (3) study the factors associated with health insurance change and with problems getting life insurance, and (4) explore the association between change in health insurance and economic well-being, of very long-term (14–24 years post-diagnosis) cancer survivors in Germany.

## Methods

### Setting and participants

#### CAESAR follow-up study

CAESAR (CAncEr Survivorship—A multi-Regional population-based study) aimed to describe the long-term HRQOL of colorectal, breast, and prostate cancer survivors. The German Cancer Research Center (Deutsches Krebsforschungszentrum, DKFZ) conducted the study in collaboration with six population-based cancer registries in Germany (Bremen, Hamburg, North Rhine-Westphalia, Rhineland-Palatinate, Saarland, and Schleswig–Holstein). Cancer survivors diagnosed between January 1994 and June 2004 as registered in the participating cancer registries, and aged between 20 and 75 years at diagnosis were eligible. Details of the initial study have been described elsewhere (Arndt et al. 2017). Initial recruitment was in 2008–2011. Between 2018 and 2019, a follow-up assessment was conducted among survivors who had given consent at initial recruitment to be re-contacted and who were still alive.

The ethics committee of the University of Heidelberg and the responsible local ethics committees of the participating cancer registries approved the initial and follow-up studies. All participants provided written informed consent.

### Data collection

Relevant data from 2008/2011 and 2018/2019 were combined for this analysis.

#### 2008/2011 wave

##### Demographics and clinical data

Self-reported data include education, marital status, treatment received, disease recurrence since index cancer (recurrence, metastasis, new primary cancer), and comorbid conditions. Participating cancer registries provided clinical data such as date of diagnosis and the tumor stage. Classification of cancer site was according to the International Classification of Diseases—10 codes.

#### 2018/2019 wave

##### Insurance

Respondents completed questions on their current health insurance coverage. Options included were: statutory, private, supplementary private, civil service scheme (‘Beihilfe’), self-payment, state-aided, and no insurance. Respondents were also asked whether they had changed their health insurance provider since their cancer diagnosis, and if yes, whether it was between sHI, from sHI to pHI, pHI to sHI or between pHI. Respondents were also asked whether there were any problems getting health insurance since cancer diagnosis and if yes, how were these problems resolved; accepted, accepted with extra contribution, not accepted, accepted at another insurance provider.

Respondents were asked whether they had problems getting life insurance since cancer diagnosis and if yes, how were these problems resolved; accepted, accepted with extra contribution, not accepted, accepted at another insurance provider.

#### Economic well-being

Financial impact of cancer was assessed with the financial problem item from the European Organization for Research and Treatment of Cancer Quality of Life Core-30 (EORTC QLQ-C30) questionnaire (Aaronson et al. 1993). The raw score was linearly transformed to a scale of 0–100 using standard procedures (Fayers et al. 1995). Higher scores indicated greater financial problems.

### Statistical analyses

Percentages of types of health insurance coverage and changes in health insurance provider were calculated. As the majority of the samples were covered under sHI, we limited our analyses on changes in health insurance to this group. Using logistic regression, we derived odds ratios (OR) and 95% confidence intervals (95% CIs) of factors that were associated with change to another sHI provider among survivors who were currently insured with sHI. These models were adjusted for sex and age at diagnosis, where appropriate. We ran logistic regression models to identify factors associated with problems with life insurance. However, these models were not adjusted for potential confounders due to the small numbers of survivors who reported problems.

We used linear regression to derive the least square mean of financial problems according to change in health insurance. This model was adjusted for age at survey, sex, and cancer type.

## Results

In total, 2714 (breast cancer: 1237, colorectal cancer: 508, prostate cancer: 969) survivors participated in the follow-up study Table 1). The mean time since diagnosis was 16.5 ± 2.1 years and mean age at survey was 75.2 ± 8.7 years. Sixty-six percent of the survivors were diagnosed with stage I/II disease, 56% had one or more comorbid conditions, 46% had < 9 years of formal education, 74% were in a partnered relationship, and 89% were not currently employed. Non-respondents were older at diagnosis and less likely to be diagnosed with stage I disease (Doege et al. 2021).

**Table 1:** Sample characteristics

	*n* = 2714 (%)
Sex	
Female	1444 (53)
Male	1270 (47)
Cancer type	
Breast	1237 (46)
Colorectal	507 (19)
Prostate	970 (36)
Time since diagnosis (± SD)	16.5 ± 2.1
Mean age at diagnosis (± SD)	58.7 ± 8.8
Age at diagnosis	
< 55 years	750 (28)
55–64 years	1212 (45)
65 years and above	736 (27)
Missing	16 (1)
Mean age at survey (± SD)	75.2 ± 8.7
Age at survey	
< 65 years	365 (13)
65–74 years	650 (24)
75 years and above	1699 (63)
Cancer stage	
I	693 (26)
II	1064 (39)
III	214 (8)
IV	62 (2)
Missing	681 (25)
Chemotherapy	
Yes	1009 (37)
No	1500 (55)
Missing	205 (8)
Radiotherapy	
Yes	1486 (55)
No	1106 (41)
Missing	122 (5)
Comorbid conditions	
None	1179 (43)
One	878 (32)
Two or more	646 (24)
Missing	11 (0.4)
Education	
≤ 9 years	1260 (46)
10–11 years	599 (26)
12 years or more	719 (26)
Missing	36 (1)
In partnered relationship	
Yes	2018 (74)
No	650 (24)
Missing	46 (2)

	*n* (%)
Current employment status	
Full-/part-time/freelancer	264 (10)
Unemployed/retired	1982 (73)
Housewife/man	282 (10)
Others	167 (6)
Missing	19 (1)
Current health insurance	
Statutory	2206 (81)
Private/self-pay	389 (14)
Civil service scheme	106 (4)
‘Beihilfe’	
Social agency	2 (0.1)
Missing	11 (0.4)

Percentages might not add up to 100% due to rounding of decimal

All respondents had health insurance coverage. Most were currently covered by sHI (81%). A proportion of survivors had pHI (15%) and 4% were covered by health insurance available to members of the German civil service and immediate family (‘Beihilfe’). The majority (93%) of the survivors reported no change in their health insurance (Supplementary Fig. 1). Of the 174 (6%) who reported a change in health insurance provider, most (*n* = 151, 86%) changed between different sHI providers, 15 (9%) from sHI to pHI, and 8 (5%) from pHI to sHI.

### Health insurance

#### Factors associated with change in sHI provider

In unadjusted analyses, being < 55 years at diagnosis was associated with increased odds of changing sHI provider (Table 2). On the other hand, factors associated with reduced odds of change in sHI provider were having colorectal or prostate cancer, being male, and being ≥ 65 years at time of diagnosis. After adjustments of age at diagnosis and sex (where appropriate), the odds for changing sHI among prostate cancer survivors increased to 2.79 (95% CI 1.01–7.68) but the association with colorectal cancer became non-significant. Being ≥ 65 years at time of diagnosis (OR_adjusted_: 0.58, 95% CI 0.35–0.96) and having ≥ 2 comorbid conditions (OR_adjusted_ 0.61, 95% CI 0.40–0.92) were associated with significantly lower odds of changing sHI provider.

**Table 2:** Factors associated with change to other statutory health insurance providers in cancer survivors with statutory health insurance (*n* = 151)

	*n*	OR_unadjusted_ (95% CI)	OR_adjusted_^a^ (95% CI)
Cancer type			
Breast	91	1.00	1.00
Colorectal	23	0.62 (0.38–0.97)	1.15 (0.66–2.03)
Prostate	37	0.58 (0.39–0.86)	2.79 (1.01–7.68)
Sex			
Female	107	1.00	1.00
Male	44	0.52 (0.36–0.74)	0.79 (0.53–1.19)
Cancer type by sex			
Female			
Breast	91	1.00	1.00
Colorectal	16	0.98 (0.56–1.71)	1.18 (0.67–2.09)
Male			
Prostate	37	1.00	1.00
Colorectal	7	0.55 (0.24–1.26)	0.46 (0.20–1.10)
Stage at diagnosis			
I	46	1.00	1.00
II	53	0.75 (0.50–1.13)	0.95 (0.62–1.46)
III	15	1.04 (0.57–1.92)	1.21 (0.65–2.26)
IV	2	0.50 (0.11–2.13)	0.81 (0.18–3.55)
Missing	35		
Age at diagnosis (years)			
< 55	72	1.73 (1.10–2.71)	1.55 (0.98–2.47)
55–64	57	1.00	1.00
≥ 65	22	0.54 (0.33–0.91)	0.58 (0.35–0.96)
Comorbid conditions			
None	57	1.00	1.00
One	49	0.84 (0.57–1.25)	0.82 (0.55–1.22)
Two or more	45	0.68 (0.45–1.01)	0.61 (0.40–0.92)
Education			
≤ 9 years	76	1.00	1.00
10–11 years	43	1.12 (0.76–1.65)	0.90 (0.60–1.34)
≥ 12 years	32	1.13 (0.74–1.74)	0.97 (0.62–1.52)

*OR* odds ratio, *CI* confidence intervals

^a^Adjusted for age at diagnosis and sex, where appropriate

#### Problems with changing between sHI providers (data not shown)

Only six (4%) survivors who changed between sHI providers reported difficulties with the change; two were eventually accepted, one was accepted with additional contribution, one was not accepted, one was accepted at another provider, and one result not indicated. Of the 15 survivors who reported changing from sHI to pHI, 3 reported having problems which were resolved with additional contribution. Of the eight survivors who changed from pHI to sHI, only one reported having problem which was resolved with additional contribution.

#### Health insurance change and financial problems due to cancer

There were no significant differences in the adjusted mean financial problems score between survivors who changed sHI providers with those who did not (15.5 versus 12.8, *p* value = 0.16).

### Life insurance

Three hundred and ten survivors (11%) indicated they had bought life insurance after cancer, of whom 25 (8%) reported having had problems (Supplementary Fig. 2). The majority (79%) had their request rejected, 8% were eventually accepted or against higher premium, and 4% was accepted at another insurance provider. Having ≥ 12 years of education (unadjusted OR 6.45, 95% CI 2.03–20.55) was associated with higher odds for problems getting life insurance (Supplementary Table 1).

## Discussion

In this German population-based study, all cancer survivors 14–24 years post-diagnosis had health insurance, with the majority covered under sHI. Most survivors did not change health insurance providers and those who did, changed mainly between sHI providers. Prostate cancer survivors were more likely to change sHI providers but older survivors and survivors with multi-morbidity were less likely to switch sHI providers. Survivors had minimal problems with changing health insurance providers and most difficulties were resolved with additional contribution. Only a small proportion of survivors who bought life insurance encountered problems, of whom the majority had their request rejected.

In total, 6% of the sample changed health insurance providers since their cancer diagnosis. A possible explanation is that participants in our study are older. We found that survivors who were ≥ 65 years at diagnosis and with multi-morbidity were less likely to change sHI. This is in line with studies of the general population where older age and chronic illness were associated with less likelihood of change in health insurance plans (Lako et al 2011; Pilny et al. 2017). Furthermore, we included survivors who were diagnosed between 1994 and 2004 while the sHI market was only liberalized in 1996. Therefore, it is conceivable that our sample were less likely to switch their health insurance, even though sHI providers are obligated to offer coverage without risk assessments, surcharges or waiting periods (“contracting obligation” according to §175 para. 1 sentence 2 SGB V). Furthermore, 6% of survivors in our study who changed between sHI providers is comparable to the general German population who changed sHI providers. According to the 2012 report from the German Socio-Economic Panel study, following the liberalization of the sHI market and further reforms in 2009 to standardize contribution rates and increase transparency of add-on premiums, between 5 and 10% of the population changed sHI providers (Rahmann and Schupp 2013).

Of the three cancer types, survivors of prostate cancer were most likely to switch sHI. One possible explanation could be the desire to seek more competitive health plans. Prostate cancer survivors under hormone treatment require a more intensive follow-up, when compared with breast or colorectal cancer survivors. A German study reported that the estimated 10-year follow-up costs for prostate cancer survivors has increased, in particular if hormone therapy is prescribed, from EUR 1361 in 2000 to EUR1777 in 2015, after accounting for inflation (Michaeli and Michaeli 2021). In addition, a greater portion of these increased costs were borne by survivors rather than insurance providers. Insurance providers incurred approximately one-third of follow-up costs in 2000 but coverage of these costs has reduced to approximately 20% by 2015.

In our study, only a small proportion of survivors who tried to change health insurance or buy life insurance had their request rejected. Nevertheless, the phenomenon of survivors being denied insurance coverage or getting a loan after their cancer is common. In the EU, there is a movement working towards facilitating cancer survivors’ access to financial services by ‘forgetting’ or deleting the cancer history of survivors with good prognosis and have remained cancer-free for a determined period. The European Cancer Patient Coalition started in 2020 the Right To Be Forgotten (European Cancer Patient Coalition 2020) project to assess and monitor potential obstacles cancer survivors in the EU face when accessing financial services. To this end, EU member states such as France (Dumas et al. 2017), Belgium, Luxembourg, and The Netherlands (Nederlandse Federatie van Kankerpatienten Organisaties 2021) have adopted national legislation to acknowledge the right to be forgotten.

Our results could be considered as positive because cancer survivors who tried to change sHI or buy life insurance were, in general, able to do so without difficulties. However, the situation might be different for more recently diagnosed cancer survivors (e.g., within the last 10 years). Further reforms in the German sHI system since 2000, such as the introduction of integrated care and selective contracts to increase competition and manage health costs (OECD 2016), may have encouraged change in sHI providers. Nevertheless, whether these reforms have improved the quality of care for insurance enrollees is open to discussion (Busse et al. 2017). Policy makers should be aware of resulting inequalities, conflicting with the ideal of a fair and solidarity insurance system in Germany. Future, ideally prospective, studies that include a younger sample with more recent diagnosis are recommended.

Although our study spotlights an under-researched issue, there are limitations to be considered. Despite the large sample of long-term cancer survivors, only a small proportion changed health insurance or bought life insurance after cancer. The potential of underassessment of events and potential reduced power of the study should be acknowledged. Further, we do not have the reasons behind change in health care insurance. Changes in insurance are self-reported which raises the possibility of recall bias, and we do not have administrative data from insurance providers for corroboration. For respondents who tried to get health or life insurance but were rejected, we do not know how many insurance providers they had approached. We are also no able to infer causal association on change in sHI due to the cross-sectional design.

In conclusion, most cancer survivors did not change their health insurance provider nor tried to buy life insurance after cancer diagnosis. Most problems with changing sHI were resolved with additional contribution while the main problem encountered when buying life insurance was rejection of request.

## Supplementary Information

Below is the link to the electronic supplementary material.

Supplementary file1 (DOCX 140 KB)

## Data Availability

Data used in this study are available on request.
