# Supplementary material for: Health and life insurance-related problems in very long-term cancer survivors in Germany: a population-based study
Source: J Cancer Res Clin Oncol. 2021 Oct 13;148(1):155–62. doi: 10.1007/s00432-021-03825-x (PMC8752534; doi:10.1007/s00432-021-03825-x)
Supplement: Supplementary file 1 — Supplementary file1 (DOCX 140 KB) [file 432_2021_3825_MOESM1_ESM.docx]

**Supplementary Figure 1. Proportion of change in health insurance providers**

13 respondents had missing information on change in health insurance.


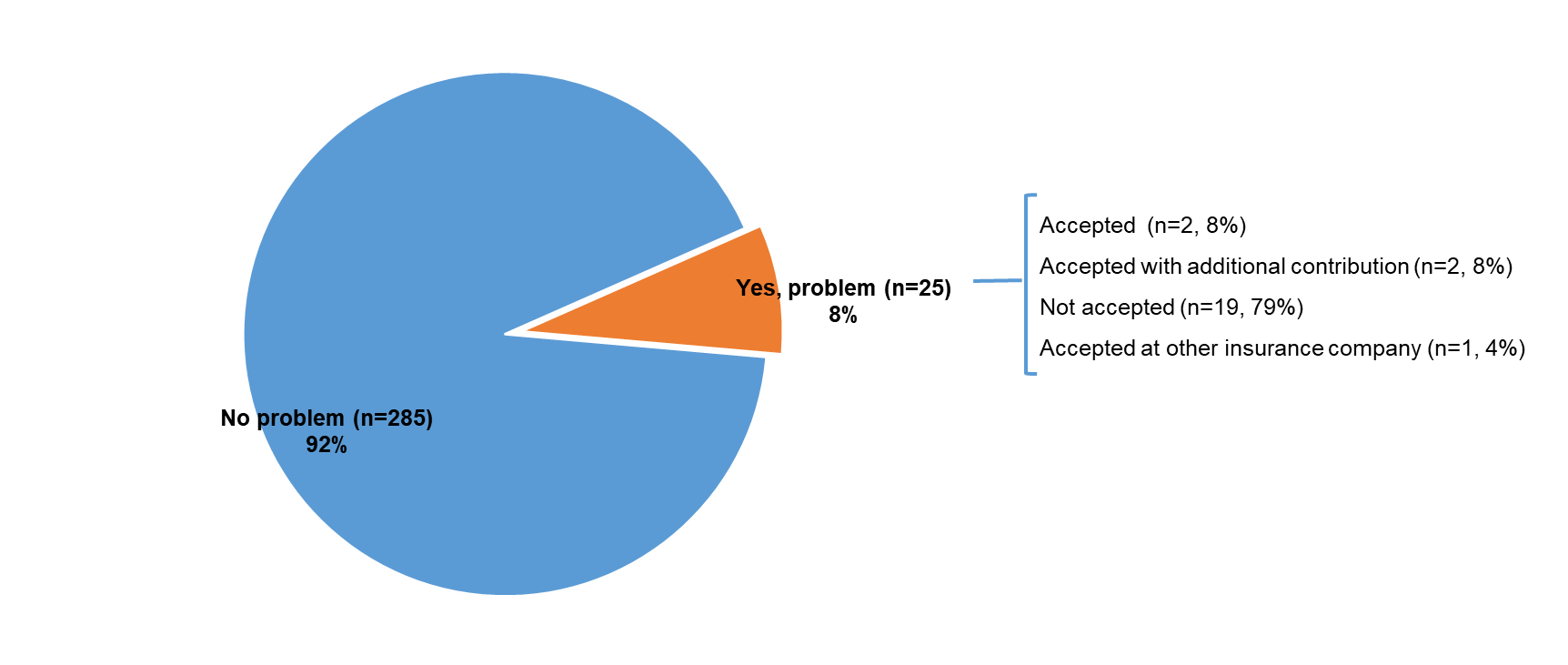


**Supplementary Figure 2. Distribution of cancer survivors who bought life insurance after cancer, encountered problems, and how problems were resolved**

Survivors who reported how problems were resolved: n=24

Percentages might not add up to 100% due to rounding of decimal

**Supplementary Table 1. Factors associated with problems obtaining life insurance (n=25)**

|  | **n** | **OR_unadjusted_ (95% CI)** |
| --- | --- | --- |
| **Cancer type** |  |  |
| Breast | 15 | 1.00 |
| Colorectal | 6 | 1.37 (0.50-3.71) |
| Prostate | 4 | 0.51 (0.16-1.59) |
| **Sex** |  |  |
| Female | 18 | 1.00 |
| Male | 7 | 0.68 (0.27-1.68) |
| **Stage at diagnosis** |  |  |
| I | 10 | - |
| II | 5 | - |
| III | 3 | - |
| IV | 0 | - |
| Low (I &II) | 15 | 1.00 |
| High (III & IV) | 3 | 1.54 (0.42-5.68) |
| Missing | 7 |  |
| **Age at diagnosis (years)** | |  |
| <55 | 17 | 2.98 (0.87-10.29) |
| 55-64 | 5 | 1.00 |
| ≥65 | 3 | 0.36 (0.12-1.10) |
| **Comorbid conditions** | |  |
| None | 8 | 1.00 |
| One | 10 | 0.79 (0.30-2.09) |
| Two or more | 7 | 0.98 (0.34-2.82) |
| **In a partnered relationship** | |  |
| Yes | 19 | 1.00 |
| No | 6 | 0.89 (0.34-2.31) |
| **Education** |  |  |
| < 9 years | 4 | 1.00 |
| 10-11years | 8 | 3.24 (0.95-11.11) |
| ≥12 years | 13 | 6.45 (2.03-20.55) |

OR: odds ratio; CI: confidence intervals
